# Molecular Aging Markers in Patients with Klinefelter Syndrome

**DOI:** 10.14336/AD.2019.0801

**Published:** 2019-08-01

**Authors:** Eva Pohl, Sina Muschal, Sabine Kliesch, Michael Zitzmann, Julia Rohayem, Jörg Gromoll, Sandra Laurentino

**Affiliations:** ^1^Institute for Human Genetics, University of Münster, 48149 Münster, Germany; ^2^Department of Clinical and Surgical Andrology, Centre of Reproductive Medicine and Andrology, University of Münster, 48149 Münster, Germany; ^3^Institute of Reproductive and Regenerative Biology, Centre of Reproductive Medicine and Andrology, University of Münster, 48149 Münster, Germany

**Keywords:** ageing, DNA methylation, Klinefelter syndrome, telomere length

## Abstract

Molecular aging markers provide the opportunity for biological age determination in humans and to study factors, such as genetic determinants, affecting the ageing process. In males with Klinefelter syndrome (KS, non-mosaic karyotype 47, XXY), which is the most common sex chromosome aneuploidy, age-related morbidity and mortality are increased, and a significantly reduced life span has been observed. The aim of this study was to investigate whether Klinefelter patients exhibit molecular signs of premature ageing. We studied, specifically, age-associated DNA methylation patterns (by pyrosequencing) and relative telomere length (TL; by quantitative polymerase chain reaction) in blood in a cohort of Klinefelter patients (n=178 and 266 for DNA methylation and TL, respectively) aged 18-71 years and compared them to the data of age-matched healthy male (n = 184 and 196 for DNA methylation and TL, respectively) and female controls (n = 50). Age-associated DNA methylation patterns were not indicative of accelerated ageing in Klinefelter men. Significantly longer telomeres were found in the young Klinefelter subjects aged 18-24 years (mean=1.51 vs. 1.09 and 1.26 in female and male controls, respectively). However, telomere length in subsequent age groups showed no difference to controls. Gonosomal aneuploidy in Klinefelter syndrome is associated with higher baseline TL at adolescent age, but comparable TL with progressive age in other age groups.

Aging is generally regarded as a steady, uniform process of functional decline in cells and tissues. The pace of aging however, differs between individuals as it is influenced by genetic background, lifestyle, and disease [1,2]. To investigate differences in the pace of aging, molecular markers have been proposed for biological age determination.

The classical method for “biological age” assessment is the determination of genomic telomere length (TL) [3]. Telomeres become shortened during each DNA replication step, and are thus considered a readout for cellular proliferative capacity and replicative senescence [4,5]. Shortening of telomeres was found to be associated with human diseases, e.g. cancer [6], cardiovascular disease [7,8], and inflammatory pathologies [9,10].

Another biomarker of aging, the more recently introduced 'epigenetic clock', is based on age-related linear changes in DNA methylation. These changes can be employed for epigenetic age prediction [11-13]. Increased epigenetic age was observed to be associated with higher all-cause mortality [14-17], less favorable cancer outcome [18], ischemic stroke [19], and trisomy 21 / Down syndrome (DS) [20,21].

Klinefelter syndrome (KS), the most common sex chromosome aberration in males (1-2:1000 male newborns), is caused by the presence of at least one supernumerary X chromosome (most commonly 47,XXY). The diagnosis of males with KS includes small testes, infertility, increased gonadotropin and normal to low testosterone levels, and clinical symptoms of androgen deficiency such as decreased libido, impaired erectile function, anaemia and decreased bone mineral density may be observed [22,23]. Affected males have an increased risk for metabolic syndrome, with or without type 2 diabetes, causing atherosclerosis and cardio-vascular diseases via enhanced inflammatory and procoagulatory mechanisms [24]. As a consequence, life-span is reduced of up to 5.6 years [25,26].

We hypothesised that patients with KS exhibit molecular aging signatures indicative of premature aging, and therefore investigated molecular aging markers, i.e. telomere length and DNA methylation markers in affected males as well as in healthy male and female controls.

## MATERIAL UND METHODS

### Subjects

KS patients were selected from the internal database of the Centre of Reproductive Medicine and Andrology (CeRA), Münster, Germany ('Androbase') [27]. The clinical and genetic characteristics of a subset of 132 (out of 266 KS) patients and of 50 female controls were described previously [24]. Briefly, KS was diagnosed by karyotyping and only patients without mosaicism were included. 184 XY men (18-71 years) served as control group. These formed part of the FAME (Fertility and Aging in Healthy Men) study cohort. Volunteers for this healthy cohort were recruited through advertisements in local newspapers, internet, and hospitals. After an online questionnaire, participants were filtered according to set exclusion criteria (smoking, illegal drug use, medication, hospitalisation within the previous month, current or former cancer treatment, renal failure, viral infection, urogenital tract malformations and surgery, diagnosis or treatment for infertility, chromosomal alterations, and participation in clinical trials during the previous year). Selected men also underwent a thorough clinical evaluation at the CeRA outpatient clinic. Several andrological parameters were measured and biological samples were collected.

All subjects provided written informed consent to the analysis of genetic material as approved by the Ethics Committee of the University and the State Medical Board (codes 2009-164-S; 2013-255-f-S).

**Table 1:** Primer sequences used for pyrosequencing and relative telomere length determination.

Target		Sequence (5’-3’)
ASPA	forward	biotin-GGAGGAATTTATGGGAATGAGTT
	reverse	AAATAATTTTACCTCCAACCCTATTC
	sequencing	ACCCTATTCTCTAAATCTCA
ITGA2B	forward	biotin-TAAGATTTGATTTTGGTTGGGGGTTTT
	reverse	AACCTTACTCCCAAAAAACTCATTTACA
	sequencing	ACAATATACTCAATACTATACCT
PDE4C	forward	GGGTAGAGGTTTGTAGTAGGT
	reverse	biotin-AACTCAAATCCCTCTC
	sequencing	GGTAGTTATAGTATGATTAGAGT
Telomere	forward	CGGTTTGTTTGGGTTTGGGTTTGGGTTTGGGTTTGGGTT
	reverse	GGCTTGCCTTACCCTTACCCTTACCCTTACCCTTACCCT
HBG	forward	TGTGCTGGCCCATCACTTTG
	reverse	ACCAGCCACCACTTTCTGATAGG

### DNA isolation and bisulfite conversion

Genomic DNA was extracted from Ethylenedi-aminetatraacetic acid (EDTA) blood samples using the FlexiGene DNA kit (Qiagen, Hilden, Germany). DNA concentration was measured by spectrophotometry (NanoDrop ND-1000) and 350 ng were bisulfite-converted (EpiTect Bisulfite kit, Qiagen).

### Pyrosequencing and age prediction

Primers for DNA amplification and pyrosequencing (Table 1) were previously published [13]. Amplification was performed applying the FastStart Taq Kit (Roche, Mannheim, Germany). Presence of correct amplicons was checked by gel electrophoresis prior to pyrosequencing (PyroMark Q24, Qiagen). Initially, 32 blood samples served as an independent training set for obtaining an age-prediction formula (derived from [13]):

Predicted age (in years) = 67.96 - 81.09 α + 4.22 β + 109.72 γ where α, β, and γ are the methylation values for cg02228185 (*ASPA*), cg25809905 (*ITGA2B*), and cg17861230 (*PDE4C*), respectively.

### Telomere length determination

Mean relative telomere length was measured by quantitative PCR (qPCR) as described previously [28,29]. Briefly, cycle thresholds for telomeric repeats (T) and a single-copy gene (S), hemoglobin subunit gamma (HBG), were determined according to the mathematical model by Pfaffl *et al.* [30] and calculated as the ratio between T and S. A standard curve was generated using reference DNA from 10 randomly selected subjects. All measurements were carried out in triplicates.

### Statistical analysis

Statistical tests and data visualisation were performed using R 3.3.1 (R Core Team, 2016). Decision for a statistical test was based on the analysis of data distribution and homoscedasticity by Shapiro-Wilk test and Levene’s test, respectively. When no normal distribution of data could be assumed, non-parametric tests were utilised. Differences between slopes of linear curves were tested using analysis of variance (ANOVA) between two linear models.

**Figure 1.**
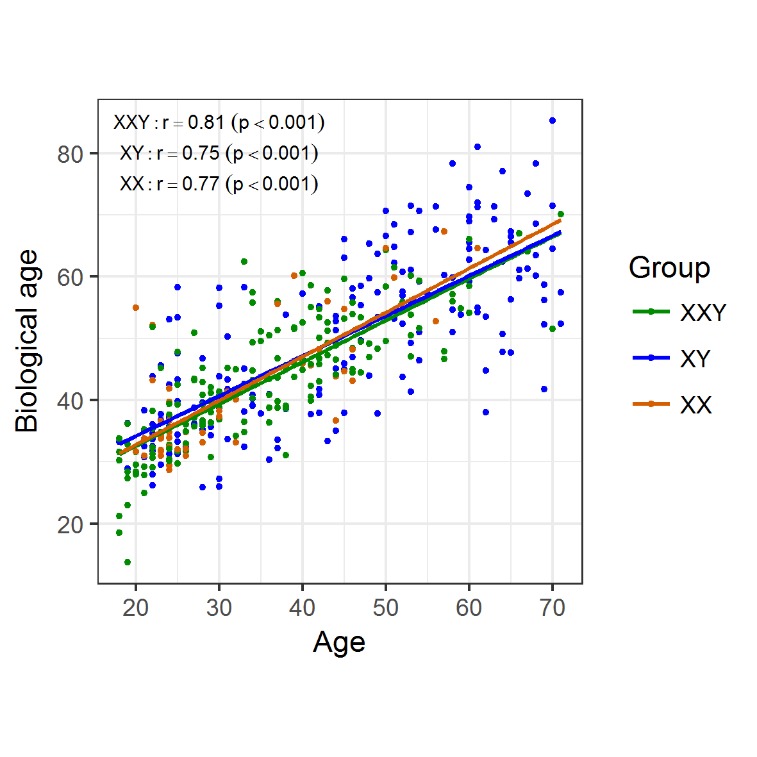
**Biological/epigenetic age was determined for individuals in the three groups and plotted against chronological age**. There was a strong positive linear correlation between the two, regardless of the group.

## RESULTS

To investigate molecular aging signatures in KS patients we determined DNA methylation in aging-associated CpG sites for molecular age prediction in blood DNA according to Weidner *et al*. [13]. To this end, we analysed 178 KS patients between 18 and 71 years of age and compared them to 184 healthy age-matched XY male and 50 female controls. For KS patients as well as the controls, we found a significant positive correlation between chronological age and predicted age (Klinefelter: r = 0.81, p < 0.001; XY males: r = 0.75, p < 0.001; females: r = 0.77, p < 0.001) (Fig. 1). We detected no differences between the slopes of the linear regression curves of both groups, which might indicate the same pace of age-dependent DNA methylation changes in patients and controls.

**Figure 2.**
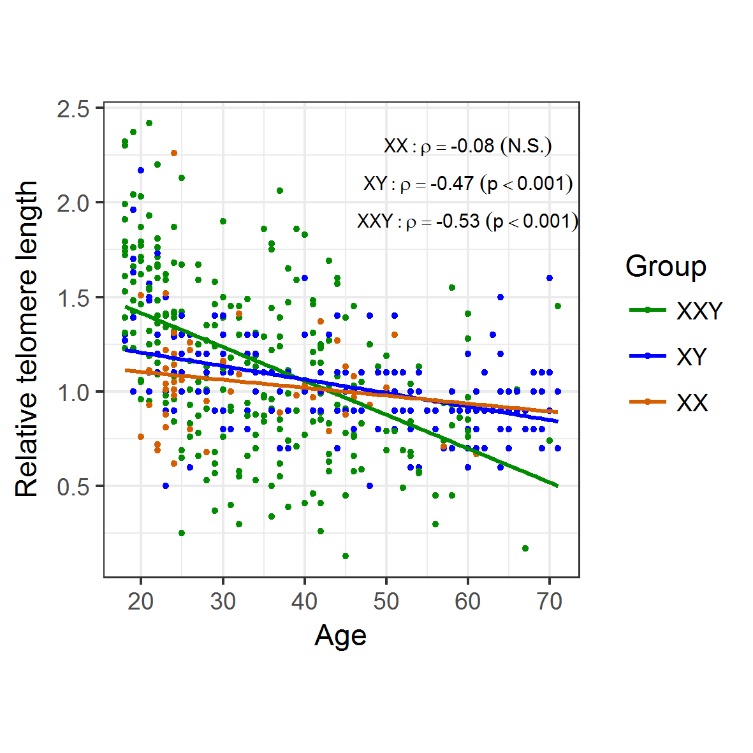
**Changes in telomere length with age, according to group**. Telomeres suffer attrition with age, regardless of genotype, however telomere shortening shows a steeper decrease in KS men (m = -0.02) compared to XY males (m = -0.007; p < 0.001) and XX females (m = -0.004; p < 0.01) probands.

**Figure 3.**
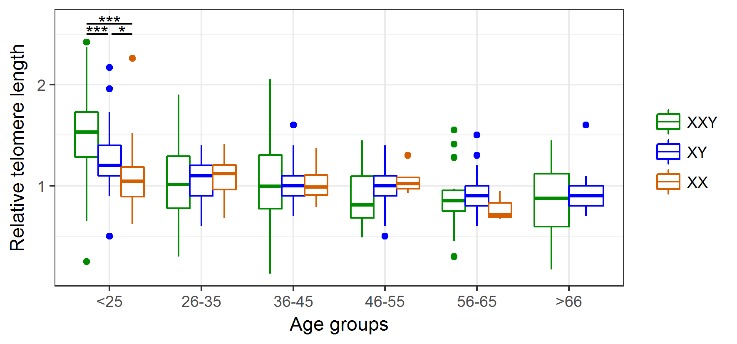
**Age-group associated changes in telomere length**. Comparison of the average rTL in the three study groups reveals that KS patients present significantly longer telomeres than both XY males and XX females only in the youngest age group. * p < 0.05, *** p < 0.001.

As the classical hallmark of aging, relative telomere length was measured in blood DNA samples of 266 patients with KS and compared to 196 healthy XY males and 50 females. We took advantage of another patient cohort, which includes 134 Klinefelter patients which were characterised in a previous study [24]. In this cohort, we detected a significant negative correlation between telomere length and age for the Klinefelter patients with ρ = −0.53 (p < 0.001) as well as the healthy XY men with ρ = −0.47 (p < 0.001). For the female control group, we only noticed a weak correlation/association with ρ = −0.08 (Fig. 2). To analyse whether there is a possible difference in telomere reduction between the groups, we compared the slopes of the linear models and found a significant difference between Klinefelter and XY men (p < 0.001) and between women and Klinefelter (p < 0.01), but not between women and XY men. In addition, we also performed an analysis of the different data subsets according to age groups (Table 2). By doing so, we found significant longer telomeres in KS patients compared to the healthy men at an age of 18-24 years (p < 0.001) (Fig. 3), although this difference faded in the subsequent age groups.

**Table 2:** Number of samples per group and age group.

Group	Age group	N (rTL)	N (Age determination)
XY	<25	42	33
	26-35	34	34
	36-45	27	26
	46-55	38	38
	56-65	38	36
	>66	17	17
XXY	<25	87	44
	26-35	64	47
	36-45	66	50
	46-55	30	22
	56-65	15	11
	>66	4	4
XX	<25	22	22
	26-35	10	10
	36-45	10	10
	46-55	5	5
	56-65	3	3
	>66	-	-

## DISCUSSION

This study is the first to describe molecular aging markers (specifically DNA methylation-based age prediction and rTL) in KS patients from late adolescence to senescence. Our hypothesis, that KS exhibits molecular aging signatures indicative of premature aging, was not supported by the epigenetic age prediction model. However, we observed striking differences between KS patients and XY men regarding telomere length at different ages: Significantly longer telomeres were present in young KS men (18-24 years) compared to healthy XY controls, followed by a steeper slope of telomere length shortening during young adulthood, leading to similar TL in middle and older aged KS subjects.

TL has been widely accepted used as a cellular marker of aging. Recently however, additional markers have been developed based on DNA methylation. These epigenetic age predictors or ‘epigenetic clocks’ show a higher correlation with age than usually obtained from the measurement of TL. These ‘epigenetic clocks’ have been used to describe accelerated epigenetic aging in various diseases and in patients with increased all-cause mortality [14-18, 20]. In contrast to these findings, we did not detect such changes in our Klinefelter patient cohort. The slight reduction in lifespan (up to 5.6 years) [26], could lead to changes in epigenetic aging that are too subtle to be detected by the applied method and sample size. Alternatively, it may be that the KS pathophysiology does not affect the measured epigenetic aging signatures. In DS the reduction in lifespan is more pronounced with a life expectancy of approximately 60 years [31] and thus the effects on DNA methylation are stronger and thereby measurable.

The finding that young KS patients exhibit longer telomeres compared to healthy controls is similar to findings in the common autosomal aneuploidy DS. Two studies showed that young DS patients (2-21 years) have longer telomeres than euploid controls [32.33]. Vaziri *et al*. proposed that there is an accelerated telomere loss in the DS patients [32]. Similarly, we found a steeper negative slope of the linear regression in Klinefelter subjects compared to the controls supports, which might support the idea of increased telomere attrition (Fig. 2). However, since our study is cross-sectional and not longitudinal, we cannot ascertain whether this difference in TL in younger ages is due to increased telomere attrition in the individual KS patients. Our analysis revealed that significant differences and increased telomere attrition were restricted to young adults (18-24 years) (Fig. 3). With respect to this observation we can only speculate on a possible mechanism. Altered proliferation of hematopoietic stem cells, in conjunction with an altered telomerase activity, is associated with accelerated telomere attrition in inflamatory disorders [10]. A similar scenario might be the case for KS patients, for which a proinflammatory status has been demonstrated [23,24].

Since we noticed similarities between the two aneuploidy conditions (KS and DS) regarding TL, we speculate whether telomere attrition is linked to abnormal chromosome segregation. Indeed, previous studies showed an association between the control of telomere length and chromosome segregation through nuclear factors mediating post-translational protein modifications, i.e. a lack of SUMO E3 ligase results in defects in chromosome segregation as well as an increase in TL [35,36].

In order to evaluate whether the additional X-chromosome accounts for longer telomeres in Klinefelter syndrome, we added the female control group. However, since telomere lengths are not similar between Klinefelter patients and women, it is unlikely that genes expressed on the additional X-chromosome contribute to this phenomenon. Although the number of female controls included in our study is rather small, this view is also corroborated by the finding that women with Turner Syndrome (X0) exhibit similar TL to controls [37].

In this study we only used two molecular markers of aging previously found to be highly informative for the determination of biological and cellular aging (for a comprehensive review see [38]). In fact, there are several ‘epigenetic clocks’, differing in the DNA methylation analysis platform employed and on the number of CpG sites analysed. The validity of the method we employed, based on the determination of DNA methylation in only 3 CpG sites by pyrosequencing, has been previously demonstrated [39,40]. In general, these ‘clocks’ show a stronger correlation with age than what is found for TL [41, 42], which is in accordance with our findings. While DNA methylation is unarguably a better measure for biological aging than TL, these two types of markers are likely measuring different features of the aging process and are therefore both of importance [43].

The present study cohort comprises clinically well-characterised subjects and consists of a relatively large sample size. However, future longitudinal studies in Klinefelter syndrome are needed to follow the progression of molecular markers at an individual level.

In summary, we evaluated molecular aging markers in KS patients and describe increased blood TL in young KS patients with normalization in later age groups, while biological/epigenetic age remains unchanged. It remains to be elucidated whether the telomere length kinetics in KS is linked to the pathophysiology of KS, its numerous comorbidities, and the reduced lifespan or to as yet unidentified factors, and also, if a period of accelerated telomere loss occurs, it might have yet unidentified consequences for the future health of KS patients.
